# Web-based database for the management of tissue specimens in a transregional histological research facility

**DOI:** 10.1186/1746-1596-6-17

**Published:** 2011-03-10

**Authors:** Sebastian Huss, Florian Fronhoffs, Reinhard Büttner, Lukas C Heukamp

**Affiliations:** 1 University Hospital, Institute of Pathology, Cologne, Germany; 2 University Hospital, Institute of Pathology, Bonn, Germany

## Abstract

**Background:**

In the setting of a histological research core facility sample tracking and project specific archiving of tissue specimens and communication of related data is of central importance.

**Description:**

Over a 24-month period 10 laboratories from two transregional research centers submitted in excess of 3000 tissue samples for histological processing and evaluation to our core facility. A web based database was set up to overcome the logistical problem of managing samples with inconsistent, duplicate and missing labels and to allow for efficient sample tracking, archiving and communication with the collaborating research laboratories. The database allows the users to remotely generate unique sample identifiers and enter sample annotation prior to sample processing. Furthermore the database facilitates communication about experimental set-up results and media files such as histological images.

**Conclusion:**

Our newly constructed web based portal is an important tool for the management of research samples of our histological core facility and facilitates significantly interdisciplinary and transregional research. It is freely available for scientific use.

## Background

**Figure 1:**
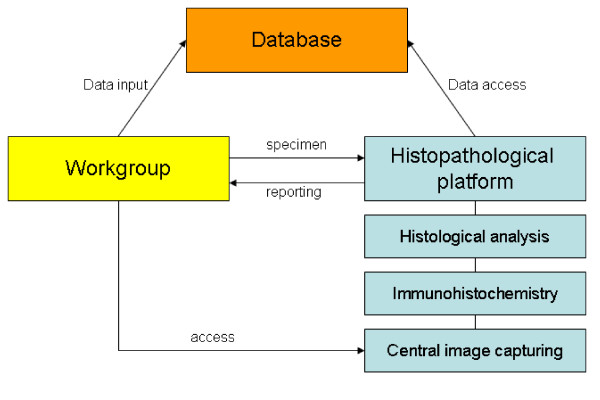
**Work flow of the histopathological platform**. The different research laboratories provide tissue samples and enter related data in the database. The histopathological platform will embed the specimen and perform histopathological and immunohistochemical staining using a standardized set of antibodies. It will also analyse and quantitate pathological changes. Central image storage can be accessed by the workgroups.

Providing standardized tissue processing, immunohistochemical staining, image generation, virtual microscopy, image storage and sample archiving are the key aims of our histopathological core facility in a transregional collaborative research center (Figure 1). Furthermore histopathological reports are requested. For example a definite pathology of tissue (i.e. lung or liver) can be assessed by different techniques. Pathologists describe changes in organ architecture subjectively or semi-quantitatively by a variety of scoring systems depending on the underlying disease causing different histological patterns.

In our setting different research groups from two transregional research centers submit hundreds of animal and pseudonymized research tissue samples per year to our histopathological core facility for examination. The mission of our first german transregional research center (Sonderforschungsbereich/Transregio 57: "Organ fibrosis: From mechanisms of injury to modulation of disease") is to identify principles of liver and kidney fibrosis and to develop new strategies to inhibit organ fibrosis or induce its regression. The second german research center (Sonderforschungsbereich 832: "Molecular basis and modulation of cellular interactions in the tumour microenvironment") aims to determine key mechanisms that cause and affect the interaction between cancer cells and their microenvironment.

Scientific data are related to each specimen (i.e. type of tissue, experimental setting, and genetic background). It is important to carefully collect these data, as different projects address different types of tissue damage and the related scientific data may be important for histopathological evaluation and reporting.

Therefore a low cost sample tracking solution that allows decentralized sample labeling and central processing for relatively small samples numbers (100s to 1000s) from several sources was required, because it turned out to be impossible to manage the wealth of data by personal communication or email alone.

Numerous commercial software solutions for computerized electronic documentation of medical data are nowadays available. In our case a clinical database (Orbis, GWI AG, Germany) and a histopathological reporting database (PathoPro, IFMS, Germany) were available but did not sufficiently fulfill the requirement to manage the research samples. Standard commercial laboratory information management systems (LIMS) packages where either to bulky or did not offer a simple web interface.

We therefore opted to generate a FileMaker based database for our specific needs. We defined the following six criteria to be met to create a useful software solution: (1) unique sample identifier (ID), (2) possibility for blinded analysis (i.e. no experimental data on slides), (3) easy and intuitive to use, (4) fully accessible via the World Wide Web, (5) high level of data security, (6) accessible from different operating systems via Web (Mac OS, Windows and Linux, portable devices and mobile phones).

## Construction and content

The database was build using the current version of FileMaker Pro (FileMaker Inc., Santa Clara, California). It is written in English but can be translated into any other language as required.

It is crucial, that submitted tissue is uniquely identifiable. Therefore we created an unambiguous sample ID for each tissue sample consisting of the prefix, in our case "A" to identify the sample to belong to the core facility of this particular transregional research group, a project ID and a sequential samples number. The resulting specific sample IDs are automatically generated for each specimen using the web interface (i.e. A30/02, A30/03 or A505/70). The separated submitting research laboratories were asked to write the IDs on the front of the tissue block. Further experimental description can also be added. However a blinded analysis of the specimens' pathology is still possible, as only the unique sample ID and no further information is written on the resulting histological slides.

**Figure 2:**
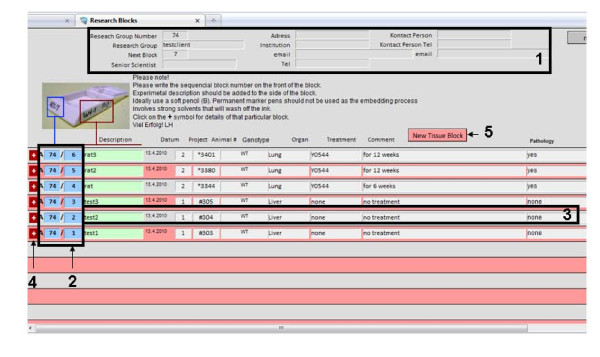
**Program interface (Main screen)**. Resolution is 1,024 × 748 pixels. On top research group related information is provided (1). On right-hand side, the sequential number of the specimen is displayed (2). There are fields for experimental description (3) that can be added to the block if appropriate. For more information a separate window may be opened (4). New data can be entered by clicking the "new tissue block" button (5).

**Figure 3:**
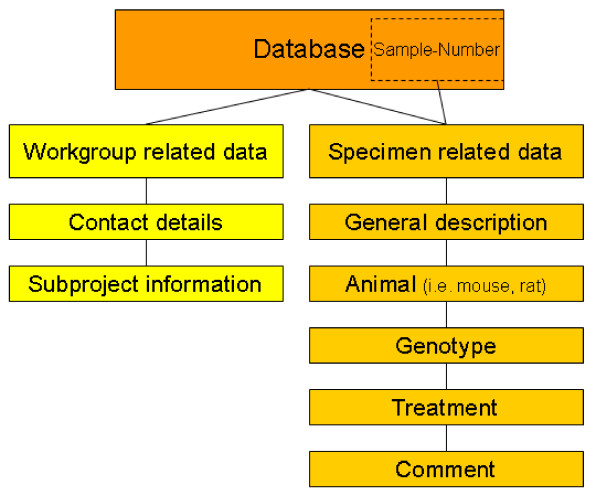
**General structure of the database**. Fields for data input: workgroup *(on left-hand side) *and specimen *(on right-hand side) *related data can be entered in the database. In example for each specimen the following data are collected: general description, project, animal type (i.e. mouse or rat), genotype, organ information, treatment and a comment if reasonable.

**Figure 4:**
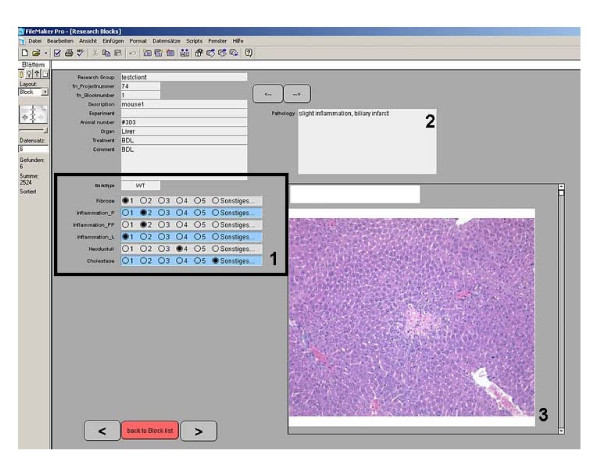
**Program interface (Sub screen)**. Resolution is 1,024 × 748 pixels. Different scoring values can directly be entered into the database (1). A comment on the general pathology (2) and different pictures (3) can be added.

On the main screen (Figure 2) of the database the unique sample ID are automatically generated for each specimen by simply clicking the "new tissue block" button. Then the following data are intuitively collected: general description, project, animal type (i.e. mouse or rat), genotype, organ information, treatment and a comment if reasonable (Figure 3). A timestamp is automatically generated. Related to every block a sub screen can be opened (Figure 4). Here different scoring values, a comment and images (JPEG, TIFF; BMP, PDF up to 4 Gb) can be added by the pathologist to report back to the researcher.

The database is published on the web via the "instant web publishing" function build into FileMaker. This was seen as critically important as external laboratories can access the database via the web quickly and easily. The access time is mainly dependant on bandwidth and 1Mbit is sufficient. Access to the different projects is managed within FileMaker.

## Utility and Discussion

**Figure 5:**
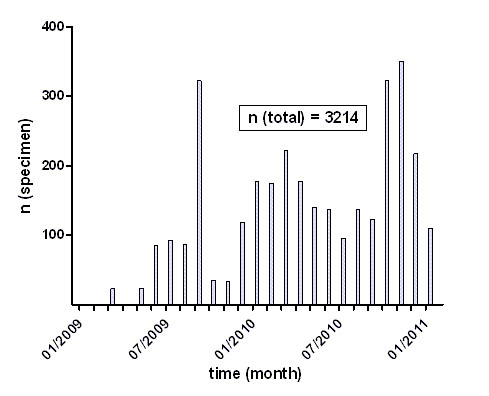
**Status report**. The graph shows the number of specimens per month entered in the FileMaker database in a 24 month period.

Over a period of two years since the establishment of the database, 10 research groups from our transregional collaborative research group and in total 3214 specimen have been registered (Figure 5). Related data are entered by external research groups and specimens were processed and evaluated by internal staff, thereby the database proved its routine usability.

We introduced the web portal in January of 2009. The users were trained by a 30 minutes session. In case of mistakes during data input, additional instruction for the corresponding scientist was arranged. Furthermore a standard operating procedure (SOP) describing the processing of the tissue specimens and the use of the database was provided on our homepage.

After a short settling-in period of several months, the database has been well accepted by our staff and coworkers and has been running without major incidence. The database has been developed with FileMaker Pro 10.0 (Santa Clara, Canada). The reasons for choosing FileMaker instead of other standard database programs (i.e. Access, Oracle or MySQL) are (1) it is easily programmable; (2) it runs in both Mac OS and Windows; (3) it has a build in web publishing engine (4) the implementation is time-saving and inexpensive. Developing a database with FileMaker is widely used with a wide range of purposes: Hambek et al. developed a database for clinical and scientific documentation of Head and Neck Oncology patients[1]. Further databases have been published in the field of cardiovascular surgery[2], electronic health care management[3], pharmacovigilance[4], parathyroid glands[5], microarray data management[6], management of mouse colonies[7] or oncological gynecology[8].

The primary goal of our database was to facilitate the work-flow in our histopathological core facility. With respect to the defined criteria to be met by our software solution (see above) the database is now useful in many different aspects: by consequent use of the specific reference number for each block data cannot be confounded and a blinded analysis of the respected tissue is possible. Furthermore by being fully accessible via the World Wide Web scientists from other cities and institutions using different operating systems on their computer can access the clearly arranged and intuitively to use database. Data security was archived by a personal login. Altogether the web portal improved the quality of the scientific documentation and has reduced time and effort of the documentation itself as well.

Concerning the future development of our database system we plan to link digitally scanned slides directly to our database. Virtual microscopy is widely accepted for educational purposes and teleconsultation and has recently gained more attention even in routine diagnostic processes [9-11]. Using a Pannoramic MIDI scanner (3DHISTECH, Budapest, Hungary) the majority of our tissue slides are digitally scanned, stored and evaluated. By a simple mouse click in our database the requested slide could be opened allowing an external researcher to (re)view these slides on his computer using a specially designed viewer software (Netbase, Freiburg, Germany).

## Conclusions

We have developed and established a scientific database system for relatively small numbers of scientific specimen (100s to 1000s) and integrated it successfully into the workflow in our histopathological core facility. The database is freely available upon request.

## Availability and requirements

We created a test client and invite our readers to access our database. The current URL, username and required password can be provided on request. In addition a free copy of the entire database is available on request.

Project name: Research Blocks Tool

Project home pages: http://www.sfbtrr57.rwth-aachen.de, http://www.sfb832.de

Operating systems: MAC/PC

Programming language: N/A

Other requirements: Filemaker 7.0 or higher

Licence: for Filemaker see http://www.filemaker.com, the database itself is freely available.

Any restrictions to use by non-academics: licence needed

## Competing interests

The authors declare that they have no competing interests.

## Authors' contributions

SH drafted the manuscript. SH, FF, RB and LCH (principle programmer), conceptualized, developed and managed the database. All authors approved and read the final version of the manuscript.
